# Current knowledge about stackable guides: a scoping review

**DOI:** 10.1186/s40729-024-00547-w

**Published:** 2024-05-31

**Authors:** Romain Lan, Camille Marteau, Chloë Mense, Frédéric Silvestri

**Affiliations:** 1 Faculté des Sciences Médicales et Paramédicales, École de Médecine dentaire, ADES, CNRS, Aix-Marseille University, 27 Boulevard Jean Moulin, Marseille Cedex 5, 13555 France; 2 Private practice, Marseille, France; 3 Department of Oral Rehabilitation, Karolinska Institute, Huddinge, Sweden

**Keywords:** Digital, Computer-guided surgery, Dental implants, Dental prosthesis, Prosthodontics

## Abstract

**Purpose:**

The rise of stereolithographic surgical guides and digital workflow, combined with a better knowledge of materials and loading principle, has enabled the placement of the temporary prosthesis at the time of implant placement. This scoping review aimed to assess the current knowledge available on stackable guides.

**Methods:**

The review focused on fully edentulous or requiring total edentulism patients. The procedure studied was the use of stackable guides for edentulous patients in order to place immediate temporary prostheses. The clinical endpoint was immediate placement of the provisional prosthesis after surgery combined with a prior bone reduction using a stackable guide.

**Results:**

12 case reports or case series articles met inclusion criteria, which did not allow an analysis by a systematic review. The included studies were case reports or case series. Most of the articles showed a base stabilized by 3 or 4 bone-pins, anchored in buccal or lingual part. Regarding the accuracy of bone reduction (ranged from 0.0248 mm to 1.98 mm) and implant placement when compared to planned, only 4 articles reported quantitative data. 11 articles showed an immediate loading with the transitional prosthesis after implant placement.

**Conclusions:**

There are as yet no prospective or comparative studies on the efficiency of this technique. In a reliable way, stackable guides seem to be able to guide the practitioner from the flap elevation to the placement of the temporary screw-retained implant supported prosthesis. Given the lack of studies in this specific field of guided surgery, further studies are needed to confirm the clinical relevance of this technique.

**Graphical Abstract:**

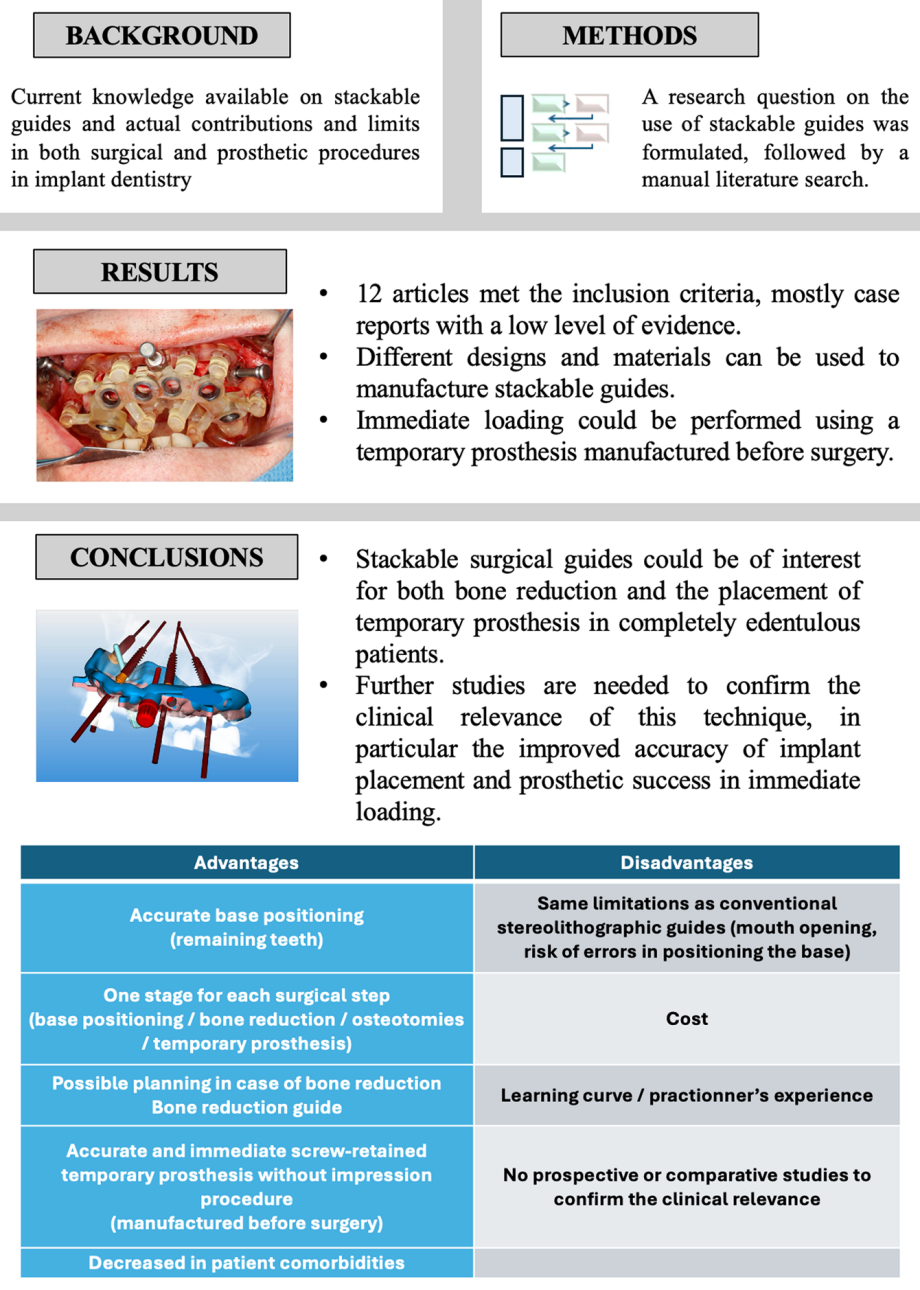

**Supplementary Information:**

The online version contains supplementary material available at 10.1186/s40729-024-00547-w.

## Background

Implant-supported screw-retained prosthesis is the gold-standard for fixed restoration in completely edentulous patient [1]. Over the past decade, digital workflow has entered the field of surgical and prosthetic implantology. Technological developments (Cone Beam Computed Tomography (CBCT), software, 3D printers, etc.) have made it possible to anticipate the production and delivery of the immediate implant-supported prosthesis. According to the literature, the rise of stereolithographic surgical guide, digital planning, and Computer-Aided Design/Computer-Aided Manufacturing, combined with improved knowledge of materials and the principle of immediate loading has led to a major advance: the delivery of a provisional prosthesis at the time of implant placement [2].

Despite an increased accuracy in implant placement compared with a free-hand surgery [3], guided surgeries could achieve surgical predictability with an average overall implant deviation of 4° from planning [2, 4]. Sometimes, this lack of precision did not allow the immediate placement of the provisional prosthesis when fabricated prior to the placement of implants. In these situations, this implied a very long session to make impressions and record the occlusion relationship following the surgery [5–7].

**Fig. 1:**
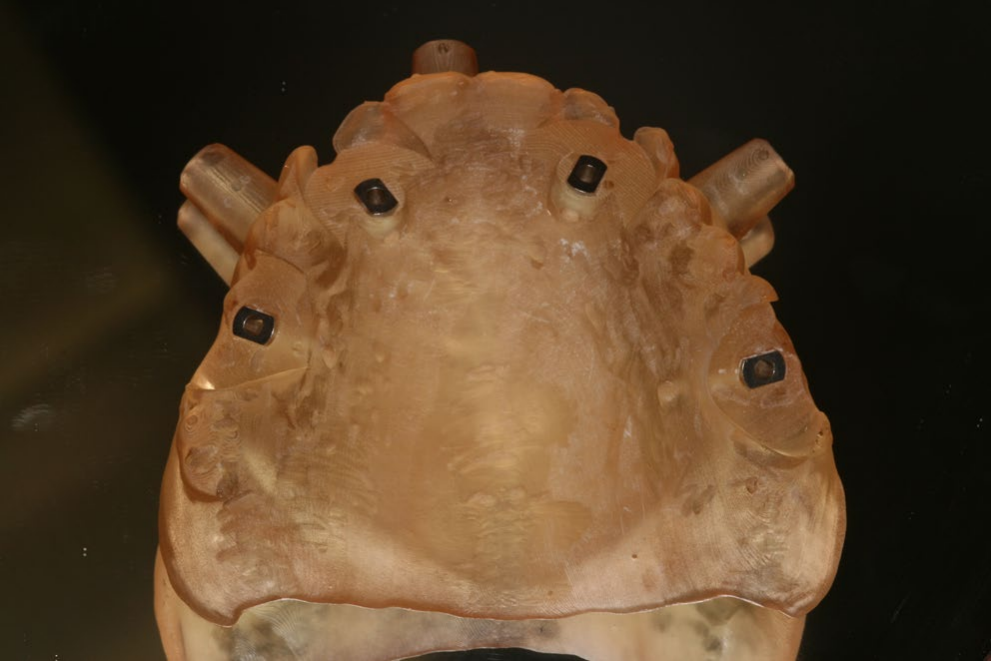
Conventional stereolithographic surgical guide (pilot drill guide)

**Fig. 2:**
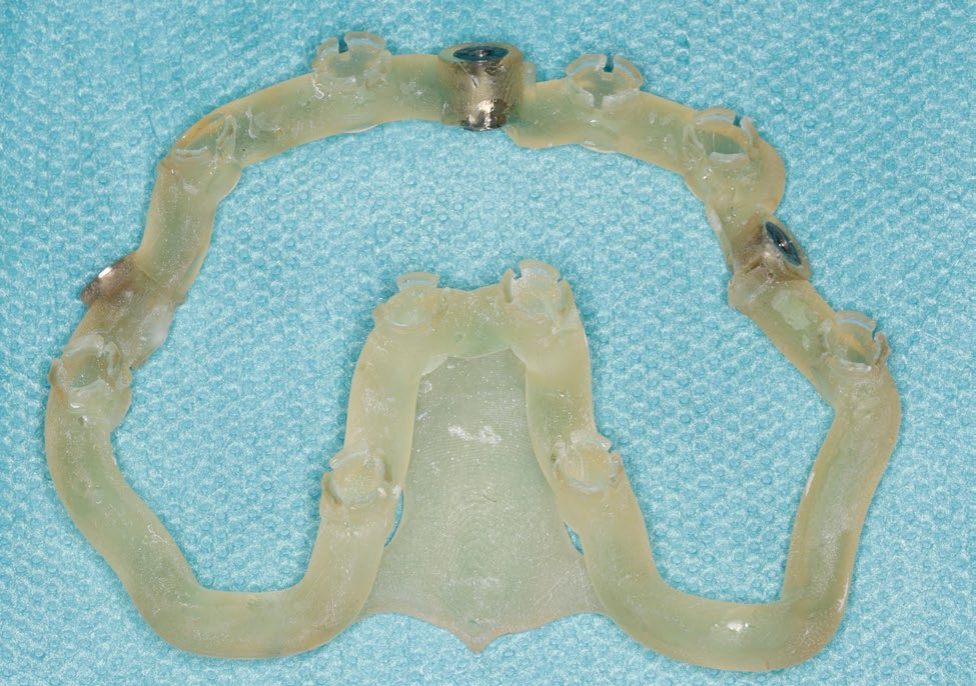
Stackable guide: base

**Fig. 3:**
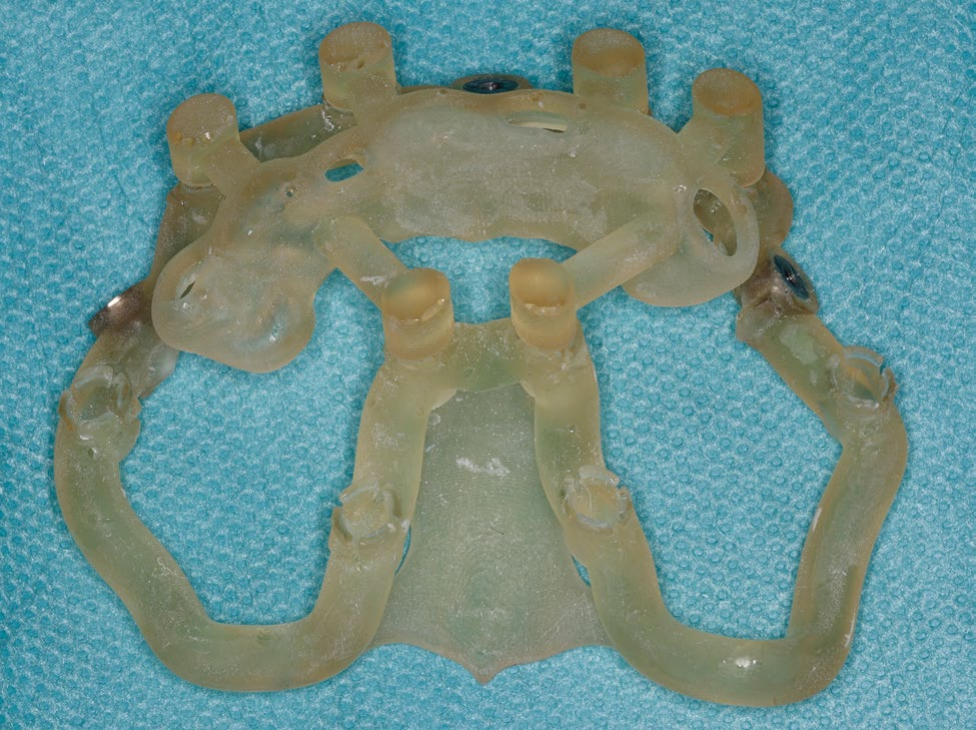
Stackable guide: base + 1rst stage (base positioning using remaining teeth)

**Fig. 4:**
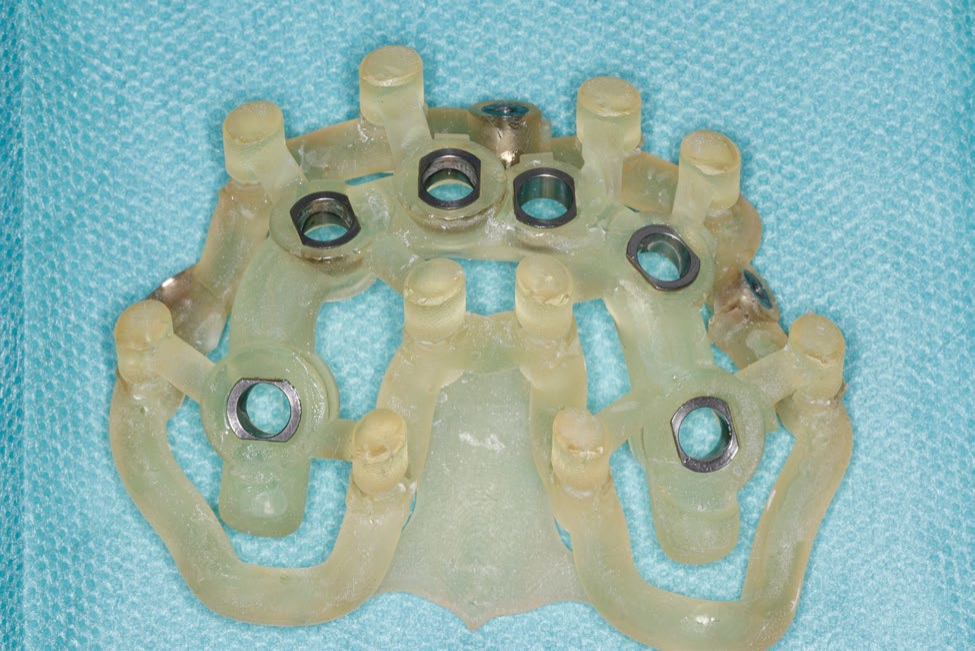
Stackable guide: base + 2nd stage for fully guided implant placement

**Fig. 5:**
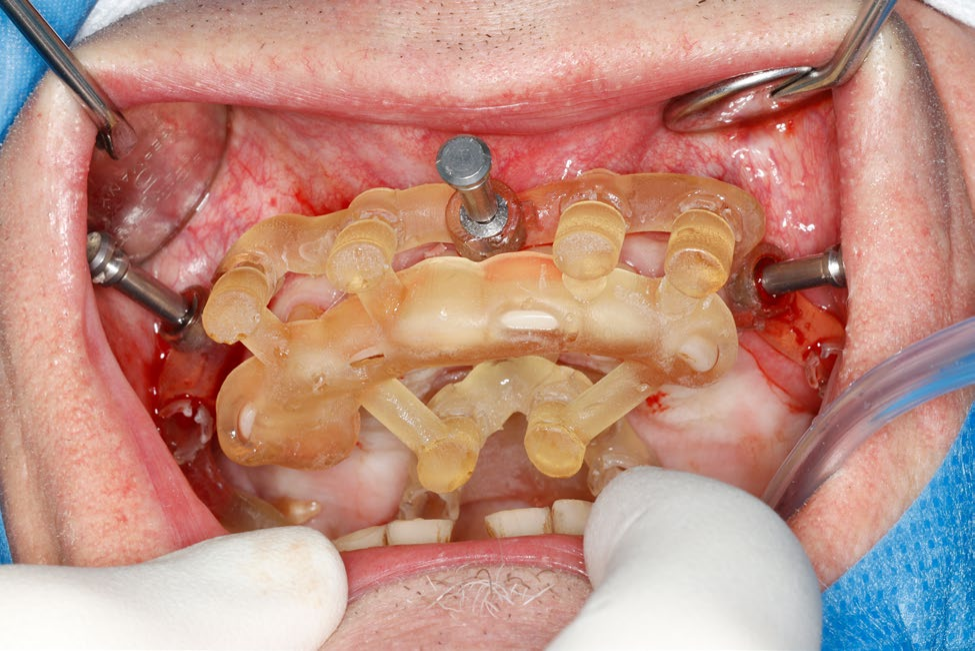
Stackable guide: base + 1rst stage (base positioning) in a clinical situation

**Fig. 6:**
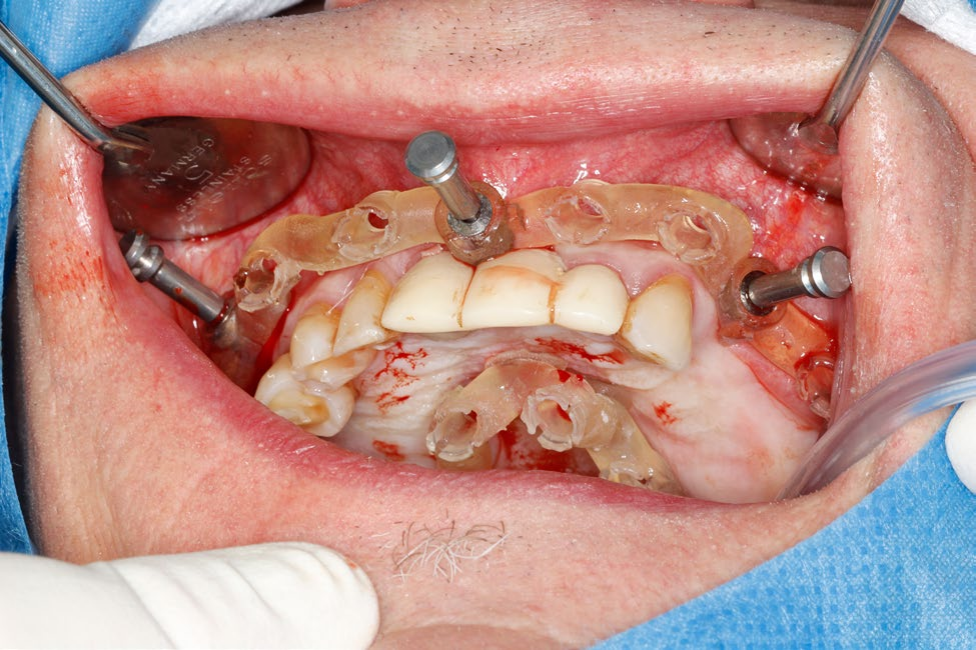
Anchored base before multiple extractions

**Fig. 7:**
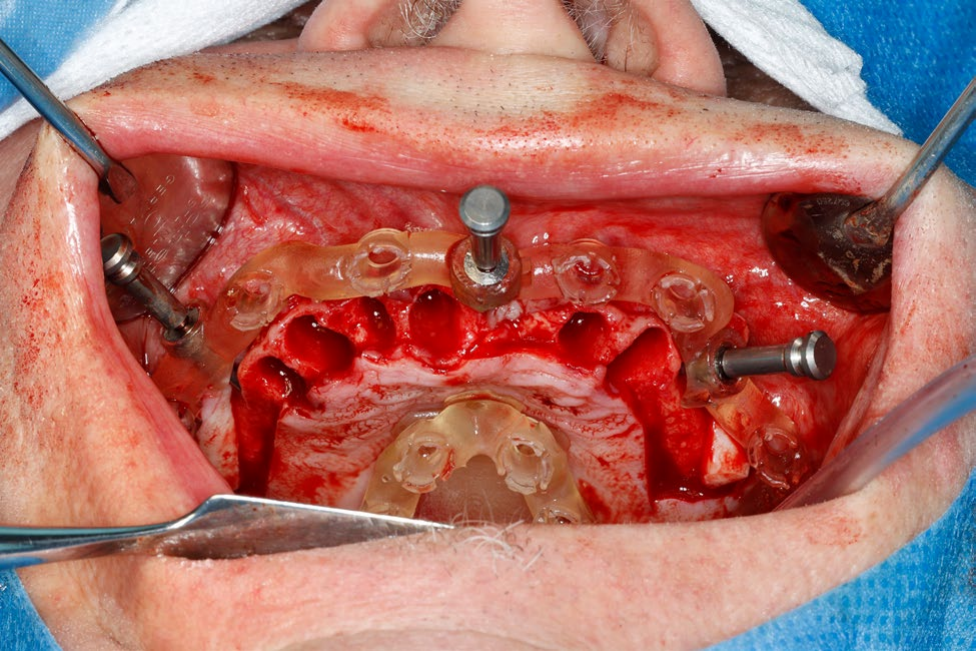
Anchored base after multiple extractions

**Fig. 8:**
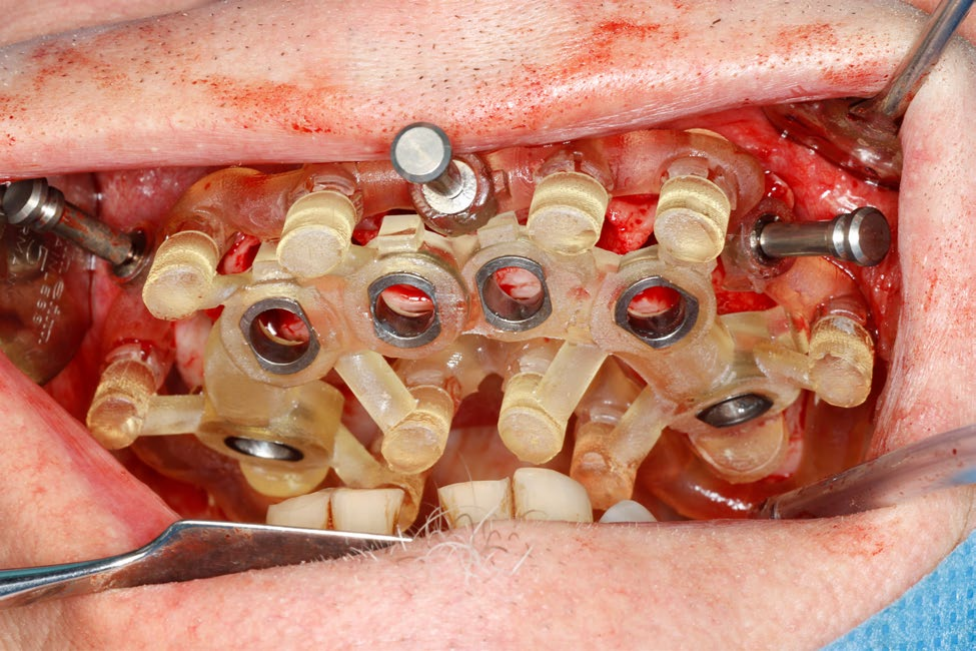
Stackable guide: base + 2nd stage for fully guided implant placement

**Fig. 9:**
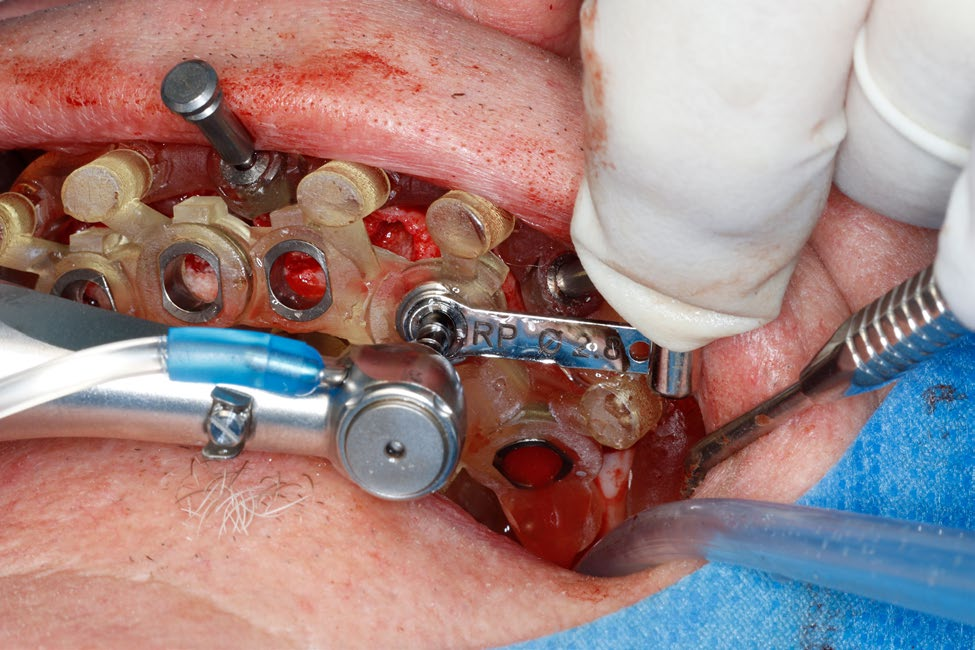
Fully guided osteotomies

**Fig. 10:**
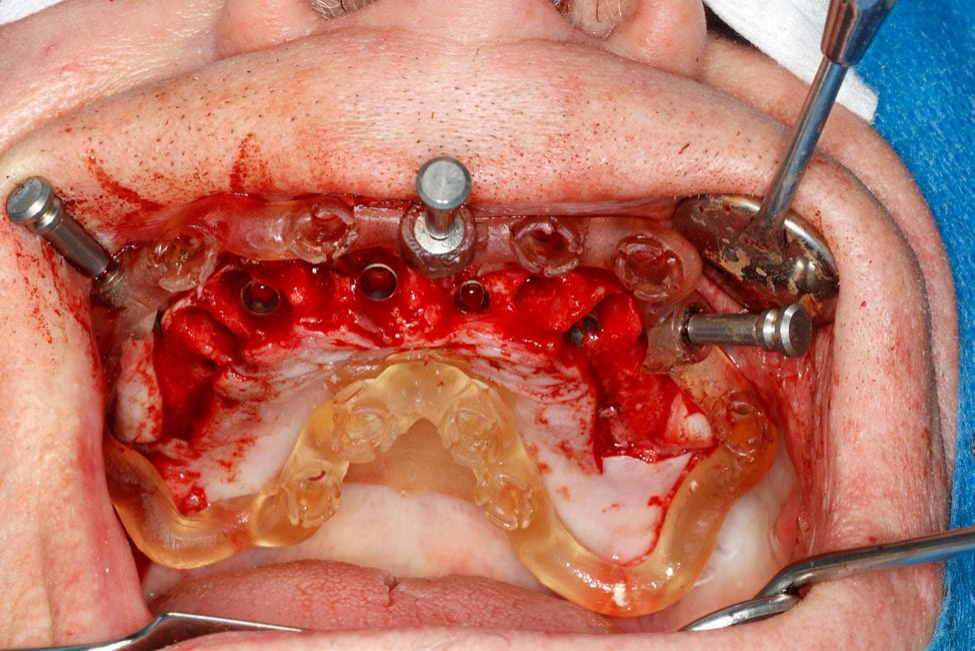
Implants placed at the end of fully guided surgery

Most of the time, in completely edentulous patients, due to an irregular post-extraction bone anatomy, guided bone reduction may be essential to establish a suitable ridge for implant placement and to fit a bone-supported drill guide [8–11]. In these specific clinical cases, conventional stereolithographic surgical guides, which are printed solely to guide drilling, cannot be manufactured to anticipate the new anatomical situation. (Fig. 1) Stackable guides are a recent evolution of stereolithographic guides whose main objectives are to achieve both bone reduction, if necessary, and placement of implants planned. The previously fabricated temporary screw-retained prosthesis can then be fitted immediately (for technical note, see Debortoli et al.) [12]. When using stackable guides, placing the bone anchored base is the first stage of a fully guided implant surgery. Different removable guides are connected with the base during the surgery to successively perform the bone reduction, osteotomies, and the placement of implants. (Figures 2, 3, 4, 5, 6, 7, 8, 9 and 10) Thus, the prosthetically-guided implant planning is followed at every stage. This type of surgical guide seems to be promising to facilitate manufacturing and the placement of a temporary prosthesis at the time of surgery, which was more difficult with conventional stereolithographic guides. In fact, the base of these guides is also used to maintain precise positioning of the provisional prosthesis while the temporary abutments are captured in the provisional prosthesis using acrylic resin.

The aim of this review is to assess the current knowledge available on stackable guides and actual contributions and limits of this device in both surgical and prosthetic procedures.

## Materials and methods

In this scoping review, the target population is completely edentulous patients or patients requiring total edentulism. All types of studies with unlimited publication period have been collected between March and May 2023 on Medline database. The procedure studied was the use of stackable guides for edentulous patients in order to place immediate temporary prostheses. The clinical endpoint was immediate placement of the provisional prosthesis after surgery combined with a prior bone reduction using a stackable guide. Other aspects of computer assisted surgery like computer guided surgery without immediate provisional placement and dynamic navigation were not studied.

Studies of partially edentulous patients whose teeth were retained or articles on freehand or non-stackable-guided surgery were not included. Animal or in vitro studies, studies published in a language other than English, and full texts not accessible via inter-university credits were excluded. Publications meeting the selection criteria were included by two independent readers according to the PRISMA methodology (PRISMA Extension for Scoping Reviews) [13].

The search started with the use of MeSH terms to obtain a search equation including the target population, the type of targeted intervention and the placement of a temporary prosthesis: “edentulous jaw AND computer aided surgery AND dental implant AND temporary prosthesis”. With this search equation, the articles obtained were too irrelevant, mostly focusing on conventional stereolithographic guides. Thus, free keywords were used in PubMed database to achieve this review: “stackable template OR stackable implant placement guide OR stackable surgical template”. This search was supplemented by a manual search in the PubMed database. General and clinical information were collected within the limitations of reported data.

## Results

We collected 12 articles that met inclusion criteria [14–25]. (Table 1)

**Table 1:** Summary of the inclusion and exclusion criteria

Inclusion criteria	Exclusion criteria
Completely edentulous jaw or indication of edentulism	Article in other languages than English
Fully guided surgery	Partially edentulous jaw
Use of a stackable guide	Another static surgical guide used or described
Bone reduction and/or temporary prosthesis	Free-hand or half-guided surgery
No year limit for publication	

The included studies were case reports (25 patients; 27 arches restored, 173 implants), corresponding to low level of evidence studies. (Tables 2 and 3)

**Table 2:** Summary of patients treated in included studies

Authors	Patients treated	Arches	Number of implants per arche	Implants	Minimum torque (*N*/cm)	Success rate in % (follow-up)
Baruffaldi (2019) [14]	11	13	6 (except one case with 8 implants in maxilla)	86	> 35	100 (> 12 months)
Berreta (2017) [15]	1	1	4	4	> 35	100 (12 months)
Costa (2020) [16]	1	1	8	8	NR	NR
Creagh (2020) [17]	1	1	8	8	NR	NR
Fu (2020) [18]	1	1	6	6	NR	NR
Garcia-Sala (2022) [19]	1	1	6	6	NR	NR
Granata (2021) [20]	1	1	6	6	NR	NR
Lanis (2021) [21]	1	1	5	5	> 50	100 (12 months)
Lu (2021) [22]	4	4	6 (2 mandibles and 1 maxilla) 8 (1 maxilla)	26	NR	NR
Papaspyridakos (2021) [23]	1	1	6	6	NR	100 (6 months)
Salama (2018) [24]	1	1	6	6	NR	100 (24 months)
Yang (2021) [25]	1	1	6	6	NR	NR

*(NR: not reported)*

- Stackable guide:

Depending on clinical cases, the guide support tissue could be different: mucosal-supported guides, bone-anchored guide, dental-supported guide and mixed-supported guide (dental and mucosal or dental and bone).

Of the 12 articles selected, regarding material used to manufacture surgical guides, only two articles reported metal alloy surgical guides, manufactured by selective laser melting [18, 25]. In the other articles, 3D printed acrylic resin was used to manufacture all the stages of stackable guides, from basis to temporary prosthesis. (Table 3)

**Table 3:** Characteristics of the stackable guides used in selected studies

Authors	About the guide								
	Number of parts	Purpose (each part)	Software	Manufacturing process	Supportive tissue	Anchoring devices	Connecting devices	Immediate loading	Immediate post-operative radiograph
Baruffaldi (2019) [14]	3	- base anchorage - drilling - interim prosthesis	CodiagnostiX, Dental Wings	3D printing (acrylic resin)	Teeth	Buccal pins (x3)	Ball attachements	Yes	Yes
Berreta (2017) [15]	2	- socle - drilling	3 Diagnosys, Ires	3D printing (acrylic resin)	Plural (teeth and bone)	Pins (x4)	Screws	Yes	No
Costa (2020) [16]	3	- bone reduction - drilling - interim prosthesis	Nemostudio software, Nemotec	3D printing (acrylic resin)	Plural (teeth and bone)	Pins	Magnets	NR	No
Creagh (2020) [17]	3	- base anchorage - drilling - interim prosthesis	Nemostudio software, Nemotec	3D printing (acrylic resin)	Soft tissue	Buccal pins (x3) Palatin pin (x1)	Notches	Yes	No
Fu (2020) [18]	3	- base anchorage - bone reduction - drilling	Materialise magics, Materialise/ Geomagic, 3Dsystem	SLM (metal alloy)	Teeth	Pins (x3)	Notches	Yes	No
Garcia-Sala (2022) [19]	3	− 4 implant removals - bone reduction − 2 implants	Meshmixer, Autodesk	3D printing (acrylic resin)	Teeth	Buccal pins (x3)	Magnet	Yes	Yes
Granata (2021) [20]	4	- base anchorage - drilling - interim prosthesis	3 Diagnosys, Ires	3D printing (acrylic resin)	Dentaire	Buccal pins (x4)	Ball attachements	Yes	No
Lanis (2021) [21]	2	- bone reduction - drilling	CodiagnostiX, Dental Wings	3D printing (acrylic resin)	Soft tissue	Pins (x3)	Notches	Yes	Yes
Lu (2021) [22]	3	- bone reduction - drilling - interim prosthesis	Blue Sky Plan, Blueskybio	3D printing (acrylic resin)	Bone	Buccal pins (x4)	Notches	Yes	No
Papaspyridakos (2021) [23]	4	- pins placement - bone reduction - drilling - interim prosthesis	CodiagnostiX, Dental Wings	3D printing (acrylic resin)	Soft tissue	Unanchored	Pins	Yes	No
Salama (2018) [24]	3	- bone reduction - drilling - interim prosthesis	Blue Sky Plan, Blueskybio/ Implant studio, 3Mespe/ Exoplan, Exocad	3D printing (acrylic resin)	Bone	Buccal pins (x3)	Screws	Yes	No
Yang (2021) [25]	4	- base anchorage - drilling - interim prosthesis	Materialise magics, Materialise	SLM (metal alloy)	Teeth	Pins (2 buccal et 1 palatal)	Screws	Yes	Yes

**(SLM: selective laser melting; NR: not reported)**

Regarding the stabilization of the guide, most of the articles showed guides with a base stabilized by 3 or 4 bone-pins, anchored in buccal or lingual part [14, 15, 17–22, 24, 25]. Only one article showed an unanchored mucosa-supported guide [23].

The number of stacked parts varied: 2 articles reported 2-part guides, 7 articles reported 3-part guides and 3 articles reported 4-part guides. When 4 stacked parts were present, the first part is tooth-supported to perform an accurate positioning of a bone-pin anchored base. This first part was then removed, and the bone resection is performed using the second part. The third part allowed full-guided osteotomies and implant placements. The temporary guided prosthesis was positioned using the last part. In case of edentulous patients, 3 or 4 stacked parts could constitute the guide. When there were two parts, a base was present on which a drilling guide or a guide for the sinus approach is positioned.

Different devices were described to connect the different parts: 5 articles described screws or pins [14, 23–26], 2 articles described ball attachments [15, 20], 2 articles described magnetic attachments [16, 19], 4 articles described notches [17, 18, 21, 22].

Regarding placement or stabilization, no problem was reported.

- Placement of the provisional prosthesis and stackable guide accuracy:

11 articles [14, 15, 17–25] reported immediate loading procedures. Regarding the accuracy of bone reduction and implant placement in relation to planning, only 4 articles reported quantitative data allowed by a superimposition of CBCTs before (surgical planning) and after the placement of implants [11, 19, 22, 23]. The difference was analyzed using a different calculation software and the data was expressed in millimeter or degrees depending on the value indicated. According to the authors, the accuracy of the actual bone reduction ranged from 0.0248 mm to 1.98 mm when compared with the reduction initially planned; one author reports a bone plane inclination of 6.03° [22]. Regarding implant placement, accuracy ranged from 0.44 mm to 1.43 mm at the implant apex, from 0.887 mm to 1.90 mm at the implant neck, and from 2.4° to 4.14° in overall implant deviation depending on the author [11, 16, 19].

## Discussion

The use of stackable guides is a developing practice. Given the lack of studies in this specific field of guided surgery, the authors concluded that a systematic review was irrelevant. The results should be noted with caution, without the possibility of recommendations or conclusions for clinical practice.

This scoping review has enabled to identify several important points, both positive and negative.

- Placement of the surgical guide:

When there are still teeth on the jaw, the authors seemed to favour anchoring the base using the remaining teeth [11, 18, 26], which is also reported in the literature to improve the accuracy of guide positioning [26–28]. When the teeth are too mobile (e.g. due to periodontal disease), a splint could be made before the CBCT to prevent any movement that would destabilize the positioning of the base [25]. An optical impression is recommended to avoid tooth movement, which would result in a distorted planning [29].

In addition, in cases of simultaneous extraction/implant placement, the dislocation movement of teeth during extraction may deform the bony tables which may complicate the placement of the guide on these modified supporting tissues. Even without bone deformation, stabilizing the guide on extraction sockets could be challenging [20]. The stackable guide provides a secure base with bone-pins and accurate placement with the dental support used prior to extractions.

In edentulous patients, attention must be paid to the compression zone between the supporting tissues and the surgical guide so as not to destabilize the latter. When the base of the stackable guide is supported by soft tissues, oedema caused by local anesthesia may lead to positioning errors. In these clinical situations, it is important to infiltrate the anesthetic solution away from the support area [14].

In cases of limited mouth opening, stacking of the guide stages may be very complicated or even impossible and the use of these guides is therefore contraindicated [24].

Although selected studies were of low level of evidence, few of them have reported data between planed and final bone reduction or implant placement [11, 16, 19, 22]. Obviously, these data can only give a trend that needs to be supported by further studies, but they seem to show small discrepancies between planning and realization.

- Stacking of the guide stages:

According to Costa et al., magnetic attachments allow for stable and reproducible stacking of guide bases [16]. The characteristics of attachment devices were not well developed in the selected studies.

- Possible planning in case of bone reduction of the ridge:

In completely edentulous patients, the drill guide will be bone-supported; if bone resection is required, a full flap is lifted so mucosal support is not possible. The surgeon will not be able to achieve an accurate free-hand bone reduction to fit the drill guide. After ridge resection, anatomical landmarks are modified, making it difficult to position the guide later [14].

The interest in guiding bone reduction is major as it would allow the use of drill guides even in patients requiring bone resection. Once the intraosseous pins of the base are placed, the reduction can be performed up to the limit of the guide, which represents bone margins planned on software before the surgery. Then a drill guide is “nested” on the base to allow a fully guided surgery from ridge reduction to implant placement. In this way, the resection is guided, and the new bone level is true to plan [11, 14, 16, 18, 21–24].

- Placement of the provisional prosthesis:

The immediate loading of the screw-retained temporary prosthesis avoids repetitive screwing/unscrewing of the implant superstructures. The provisional prosthesis is directly placed until complete osseointegration of the implants. D’Haese et al. have shown that mobilizing the implants too early during screwing/unscrewing can lead to deviation of the implant axes from the planning [30].

In addition, the prosthesis is manufactured before surgery, thus avoiding postoperative impressions which are more complex to manage at the end of surgery. When the provisional prosthesis is obtained by transforming of the temporary removable complete denture, the risk of fracture of the prosthesis is high [31]. Moreover, of the various milled materials available, polymethyl methacrylate can be used to manufacture prostheses with improved mechanical properties [29, 32].

During the digital design of the temporary prosthesis, the implant positions lead to the choice of abutment height and angulation. This step ensures the necessary passivity of the prosthesis to avoid any iatrogenic stresses on the implants during the osseointegration phase [20]. Planning is a time-consuming step, but it saves time during surgery and limits prosthetic sessions. Indeed, the temporary restoration can be placed directly after implant placement. This avoids leaving the patient without a prosthesis for 24–48 h as well as an often poorly tolerated impression and bite registration session at the end of the surgery [4, 16, 23, 28].

- Decrease in patient comorbidities:

These static guides eliminate the need for full flap surgery [33]. Indeed, it is no longer necessary to see the underlying bone structure because the guide has been designed based on a careful and accurate CBCT analysis. A flapless surgery can be performed, operating time is significantly shortened, and postoperative comorbidities decrease (bleeding, pain, oedema, hematoma) [4, 23].

Patient and surgeon comfort is improved, and the immediate loading of the implants also avoids a difficult impression session [20].

- Digital workflow:

Digital workflow is an interesting procedure that could help in treatment planning and virtual prosthetic project. Indeed, planning is done virtually on dedicated software with superimposition of DICOM files and STL files (initial situation from intraoral scanning and virtual prosthetic project). Then printing surgical templates made it possible to transfer this prosthetically guided project from the computer to the patient [34]. Several authors have reported no differences in the clinical accuracy of implant placement between additive and subtractive manufactured guides [35, 36]. In addition, acrylic resin is a suitable material for the manufacture of surgical guides, with the benefits of ease fabrication, reduced cost and less time wasted by technicians [36, 37].

There also are many advantages to this digital workflow: fewer clinical sessions are required, the provisional prosthesis fits better, and the placement of implants is more accurate [16, 38].

Limits.

- Persistent risk of error:

Despite the promising concept of stackable guides, errors can accumulate throughout treatment steps (CBCT acquisition, wax up, distortion of the planned manufacture of the guide, calibration of the 3D printer, surgical phase, anchoring the guide during surgery, stacking the different parts). The practitioner’s experience is essential in order to remain focus on the progress of each step during the surgery and be able to fix any problem or mistakes [14, 15, 19, 21].

Main limits are in the need to acquire knowledge and experience in this field; as most of new techniques, it requires a learning curve for both surgeons and dental technicians, and the need for a specific software (initial investment required).

Obviously, limits in the use of conventional stereolithographic guide are available for stackable guide (mouth opening, remaining errors in positioning the base…).

The absence of postoperative control radiography in most studies (6/12) made it impossible to objectively assess the real effectiveness of the technique presented, or the correct temporary prosthesis positioning with regard to biological or prosthetic requirements. The results should therefore be interpreted with caution.

- Financial cost:

Manufacturing the guide adds a cost to the treatment, particularly when using magnetic attachments [14, 16].

Clinical relevance:

There is no study with good levels of evidence to evaluate and measure the benefit of stackable surgery guide. However, this surgical technique appears promising in order to improve surgical precision. It differs from the use of conventional stereolithographic guides which are already used daily by several practitioners. Deep analysis and complete planning of each clinical case are time-consuming but essential steps to achieve an optimal temporary the comfort for both surgeon and patient.

In return, the comfort for both surgeon and patient is clearly increased and the surgery becomes more reproducible. (Table 4)

**Table 4:** Advantages / Disadvantages of stackable guides

Advantages	Disadvantages
Accurate base positioning (remaining teeth)	Same limitations as conventional stereolithographic guides (mouth opening, risk of errors in positioning the base)
One stage for each surgical step (base positioning / bone reduction / osteotomies / temporary prosthesis)	Cost
Possible planning in case of bone reduction Bone reduction guide	Learning curve / practionner’s experience
Accurate and immediate screw-retained temporary prosthesis without impression procedure (manufactured before surgery)	No prospective or comparative studies to confirm the clinical relevance
Decreased in patient comorbidities	

## Conclusions

There are as yet no prospective or comparative studies on the efficiency of stackable stereolithographic surgical guides, and the data found in the literature are not standardized. Only case series are reported, which makes it impossible to justify a possible impact on clinical practice.

The management of bone reduction prior to implant placement or immediate loading of a temporary prosthesis could be facilitated by using a stackable guide, which appears to be able to guide the practitioner from surgery to immediate loading of the provisional screw-retained implant-supported prosthesis.

Further studies are therefore needed to confirm the improved accuracy of implant placement and prosthetic success in immediate loading. Given the growth of dynamic guided surgeries, in-depth studies are also needed to assess the benefits of promoting this type of surgical guide.

### Electronic supplementary material

Below is the link to the electronic supplementary material.

Supplementary Material 1

## Data Availability

The datasets used and/or analysed during the current study are available from the corresponding author on reasonable request.
